# Effect of Nicotine and *Porphyromonas gingivalis* Lipopolysaccharide on Endothelial Cells *In Vitro*

**DOI:** 10.1371/journal.pone.0096942

**Published:** 2014-05-12

**Authors:** Na An, Oleh Andrukhov, Yan Tang, Frank Falkensammer, Hans-Peter Bantleon, Xiangying Ouyang, Xiaohui Rausch-Fan

**Affiliations:** 1 Department of General Dentistry II, School and Hospital of Stomatology, Peking University, Beijing, China; 2 Division of Oral Biology, Bernhard Gottlieb School of Dentistry, Medical University of Vienna, Vienna, Austria; 3 Department of Stomatology, Xuanwu Hospital, Capital Medical University, Beijing, China; 4 Division of Orthodontics, Bernhard Gottlieb School of Dentistry, Medical University of Vienna, Vienna, Austria; 5 Department of Periodontology, School and Hospital of Stomatology, Peking University, Beijing, China; University of Toronto, Canada

## Abstract

Smoking is considered a significant risk factor for both periodontal disease and cardiovascular disease (CVD). Endothelial cells play an important role in the progression of both diseases. In the present study, we investigated *in vitro* the impact of nicotine on functional properties of human umbilical vein endothelial cells (HUVECs) stimulated with lipopolysaccharide (LPS) of periodontal pathogen *Porphyromonas gingivalis*. HUVECs were stimulated with different concentrations of nicotine (10 µM-10 mM) and/or *P. gingivalis* LPS. Expression levels of intercellular adhesion molecule-1, vascular cell adhesion molecule-1, E-selectin, monocyte chemoattractant protein 1, and interleukin-8 were measured on both gene and protein levels. Cell proliferation/viability, apoptosis, and migration were also investigated. Nicotine at a concentration of 10 mM significantly decreased *P. gingivalis* LPS-induced expression of all investigated proteins after 4 h stimulation, while lower nicotine concentrations had no significant effect on protein expression with or without *P. gingivalis* LPS. Proliferation/viability of HUVECs was also significantly inhibited by 10-mM nicotine but not by lower concentrations. Migration of HUVECs was significantly decreased by nicotine at concentrations of 1–10 mM. Nicotine at a concentration similar to that observed in the serum of smokers had no significant effect on the functional properties of HUVECs. However, high concentrations of nicotine, similar to that observed in the oral cavity of smokers, inhibited the inflammatory response of HUVECs. This effect of nicotine might be associated with decreased gingival bleeding indices in smoking periodontitis patients.

## Introduction

Periodontitis is a chronic inflammatory disease which is caused by bacterial infection and leads to the destruction of periodontal tissues and resorption of alveolar bone. It is initiated by the accumulation of gram-negative bacteria in the dental biofilm [1]. Several gram-negative bacteria, such as *Porphyromonas gingivalis* (*P. gingivalis*), *Treponema denticola*, and *Tannerella forsythia*, are thought to be the primary etiological agents associated with periodontal disease [2]. Nowadays, it is widely accepted that there is an association between periodontal disease and cardiovascular disease (CVD), though no causative relationship between these diseases has been observed [3].

It is well recognized that tobacco smoking is a major risk factor for both periodontitis and CVD [3]. Smoking periodontitis patients exhibit greater bone loss, greater attachment loss, and deeper periodontal pockets than nonsmokers [4]. At the same time, smoking periodontitis patients have lower gingival bleeding indices compared to non-smoking patients, which is supposedly due to dysfunction of gingival vasculature [5], [6]. There is also strong evidence indicating that smoking causes injury to the vascular endothelium, leading to endothelial dysfunction and initiating the pathogenesis of atherosclerosis [7]–[9]. Thus, the impact of smoking on both periodontitis and CVD seems to be largely associated with its effect on endothelial cells (ECs). ECs underlie the inner surface of blood vessels and play an important role in the progression of both diseases. In particular, ECs are shown to mediate leukocyte infiltration into periodontal tissue during periodontitis [10], [11]. Endothelial dysfunction is considered to be the first inflammatory change of the vascular endothelium leading to arteriosclerosis [7]. The function of ECs in the inflammation process is associated with the production of several cytokines and adhesion molecules which regulate the migration of leukocytes toward the inflammatory area [12].

Nicotine is a major component of tobacco smoke and one of its most pharmacologically active agents. Park et al show that nicotine at concentrations similar to those found in habitual smokers does not induce any morphological changes in ECs but does enhance functional changes such as cellular proliferation, migration, and angiogenesis *in vitro* [13]. Heeschen et al suggest that nicotine enhances cellular proliferation, migration, and angiogenesis by stimulating nicotinic receptors in vascular ECs [14]. An *in vitro* study by Villablanca mentions that nicotine at concentrations observed in habitual smokers stimulates DNA synthesis and proliferation in vascular ECs, whereas higher nicotine concentrations might have cytotoxic effects [15]. Previous studies have also shown that the major periodontal pathogen *P. gingivalis* and/or its lipopolysaccharide (LPS) can activate the expression of adhesion molecules in ECs, which might then be involved in the progression of both periodontitis and CVD [16]–[18]. However, the influence of nicotine on the response of ECs to periodontal pathogens is still unknown. Therefore, in the present study, we investigated the influence of nicotine on cell proliferation, migration, and on the expression of several pro-inflammatory cytokines in ECs with and without stimulation with *P. gingivalis* LPS.

## Materials and Methods

### Cell culture

Commercially available human umbilical vein endothelial cells (HUVECs, Technoclone, Austria) were grown in endothelial cell medium (ECM) supplemented with 100 U/ml penicillin, 100 µg/ml streptomycin, 0.25 µg/ml fungizone, 2 mM L-glutamine, 5 U/ml heparin, 30–50 µg/ml endothelial cell growth supplement, and 20% fetal calf serum (FCS)^¶^. Cells were cultured in culture flasks coated with 0.2% gelatine at 37°C in a humidified atmosphere of 5% CO_2_ and 95% air. All experiments were performed using cells between the third and sixth passage and were repeated in triplicate.

Commercially available ultrapure *P. gingivalis* LPS (Invivogene, San Diego, CA, USA) was used in the present study. As reported by another study [19], LPS preparations were free from contaminating lipoproteins.

### Cell proliferation/viability

MTT assay was used for determining cell proliferation/viability. For each experiment, 2×10^4^ cells were added to each well in standard 24-well gelatin-coated tissue culture plates and stimulated with different concentrations of nicotine and/or *P. gingivalis* LPS. After incubation for 4 h, 24 h, 48 h, and 72 h, a 5 mg/ml concentration of 3-(4,5-dimethylthiazol-2-yl)-2,5-diphenyl-tetrazolium bromide (MTT solution, Sigma, St-Louis, USA) in PBS was added to each well, and culture plates were incubated at 37°C for 4 h. The medium was removed, and 500 µl dimethylsulfoxide (DMSO) was added to each well followed by a 5-min incubation on a shaker. Finally, 100 µl of each cultured solution was transferred to a separate 96-well plate, and the optical density (OD) was measured at 570 nm with an ELISA Reader (SpectraMax Plus 384, Molecular Devices, USA).

### Production of pro-inflammatory mediators by HUVECs

HUVECs were seeded in gelatin-coated 6-well tissue culture plates at a density of 5×10^5^ cells per well in 3 ml of ECM. After 24 h, the culture medium was replaced by ECM medium containing 5% FCS, and cells were stimulated with different concentrations of nicotine (10^−5^-10^−2^ M) in the presence or absence of *P. gingivalis* LPS (1 µg/ml) for 4–72 h. Non-stimulated cells were used as a negative control. After stimulation, the gene-expression levels of intercellular adhesion molecule-1 (ICAM-1), vascular cell adhesion molecule-1 (VCAM-1), E-selectin, interleukin-8 (IL-8), and monocyte chemoattractant protein-1 (MCP-1) were analyzed by real-time PCR. In addition, the cell surface expression levels of ICAM-1, VCAM-1, and E-selectin as well as the quantities of IL-8 and MCP1 protein in conditioned media were analyzed by flow cytometry and ELISA, respectively.

Real-time PCR (qPCR) was performed as described previously [20]–[22]. Isolation of total cellular mRNA and its subsequent transcription into cDNA was performed using the TaqMan Gene Expression Cells-to-CT kit (Ambion/Applied Biosystems, Foster City, USA). Real-time PCR was performed on an Applied Biosystems Step One Plus real-time PCR system using TaqMan gene expression assays (all from Applied Biosystems, Foster City, USA) with the following ID numbers: ICAM-1: Hs00164932_m1, VCAM-1: Hs00365486_m1, E-selectin: Hs00174057_m1, IL-8: Hs00174103_m1, MCP-1: Hs00234140_m1. Triplicate qPCR reactions were prepared for each sample. During the reaction run, the point at which the PCR product was first detected above a fixed threshold (cycle threshold (CT)) was recorded for each sample. Data were presented as the relative amount of mRNA in one sample versus a control (fold change) using the formula 2^(−ΔΔCT)^, meaning we used the difference between the CT of a gene of interest and the CT of the housekeeping gene GAPDH for one sample (ΔCT) and then compared this value to that of the calibrator (control) sample (ΔΔCT).

Analysis of the cell surface expression levels of adhesion molecules in HUVECs was performed using a flow cytometer (FACSCalibur, BD Bioscience, CA, USA) [21]. Cells were stained with one of the following monoclonal antibodies conjugated with phycoerythrin (all from eBioscience, San-Diego, CA, USA): mouse anti-human ICAM-1 antibody (Cat. Nr. 12-0549), mouse anti-human VCAM-1 antibody (Cat. Nr. 12-1069), mouse anti-human E-selectin antibody (Cat. Nr. 12-0627), and isotype control antibody (Cat. Nr. 12-4714). Cell counting was limited by 10,000 events.

Commercially available ELISA kits (Hoelzel Diagnostika, Cologne, Germany) were used for measurements of IL-8 (Cat. Nr. 950050192) and MCP-1 (Cat. Nr. BOS-EK0441) levels in the conditioned medium. The samples were diluted by a ratio of 1∶20 and 1∶5 for the measurements of IL-8 and MCP-1, respectively.

### Quantification of apoptosis in HUVECs by flow cytometry

HUVECs were seeded in 6-well plates and stimulated with nicotine as described above for 4-72 h. After incubation, apoptosis was detected using the Annexin V-FITC Apoptosis Detection Kit I (BD Biosciences, San Jose, CA) according to the manufacturer's protocol. Cells were stained with both annexin V and propidium iodide to detect early and late apoptosis, respectively. The cells were washed twice after staining and acquired by a flow cytometer (FACSCalibur, BD Bioscience, CA, USA). Cell counting was limited by 10,000 events. The proportion of apoptotic and viable cells was quantified using CellQuest Software (BD Bioscience, CA, USA). The standard error of the mean was calculated from four independent trials.

### Migration assay

Cell migration was assessed in a 48-well microchemotaxis chamber (Neuroprobe, Gaithersburg, MD, USA) on a polycarbonate filter with 8-µm pore size, as described previously [23]. The chamber consisted of acrylic top and bottom plates, each containing 48 matched wells. Top and bottom plates were separated by a polycarbonate filter with 8-µm pore size (Neuroprobe, Gaithersburg, MD, USA). Twenty-six microliters of serum-free medium containing different concentrations of nicotine and/or *P. gingivalis* LPS were filled in wells of the bottom plate. Subsequently, the bottom plate was covered with a filter, and the top plate was applied so that each well corresponded to that of the bottom plate. A cell suspension containing 1×10^4^ cells in 50 µL of serum-free medium was added to each well of the top plate, and the whole chamber was incubated at 37°C in humidified air with 5% CO_2_ for 8 h. After incubation, cells on the upper surface of the filter were removed over the wiper blade, and the filters were then fixed with methanol and stained with Hemacolor for microscopy (Merck, Darmstadt, Germany). The cells that migrated across the filter were counted under a light microscope at high-power magnification (x100) to measure transmigration in each well. Four fields were counted in each well, and the total number was calculated. Four wells were used for each group, and experiments were repeated in triplicate.

### Statistical analysis

All experiments were performed in triplicate. A one-way analysis of variance (ANOVA) with Tukey's HSD test was performed to assess the significance. Values of p<0.05 were considered statistically significant. All data analysis was performed using specific software (SPSS 19.0, SPSS Inc., Chicago, IL, USA).

## Results

### Effect of nicotine and *P. gingivalis* LPS on proliferation/viability of HUVECs

**Figure 1:**
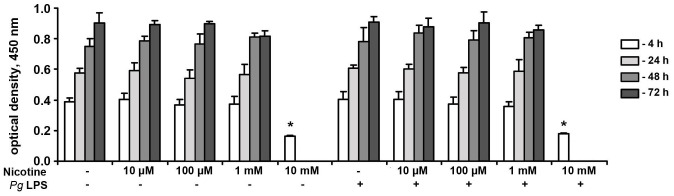
Effect of nicotine and *P. gingivalis* LPS on the proliferation/viability of HUVECs. HUVECs were stimulated with nicotine (10 µM-10 mM) and/or *P. gingivalis* LPS, and the proliferation/viability was measured after 4, 24, 48, and 72 h using the MTT assay. The y-axis represents mean ±SD values of optical densities measured at 450 nm from four wells of one representative experiment. * – significantly lower compared to control group, p<0.05.

The effect of nicotine at concentrations of 10 µM-10 mM on the proliferation/viability of HUVECs in the presence or absence of *P. gingivalis* LPS, as measured by MTT assay after 4, 24, 48, and 72 h of stimulation, is shown in Figure 1. Nicotine at a concentration of 10 mM significantly inhibited proliferation/viability of HUVECs after 4 h of stimulation. Microscopic observation revealed that after stimulation with 10-mM nicotine for 24–72 h, most cells were detached from the well bottom, and therefore no parameters were measured in these groups. No significant effects were observed at nicotine concentrations of 10 µM-1 mM for any of the stimulation time points. In all groups, except those stimulated with 10-mM nicotine, the proliferation/viability continuously increased with time. *P. gingivalis* LPS at a concentration of 1 µg/ml did not affect the proliferation/viability of HUVECs at any of the stimulation time points.

**Figure 2:**
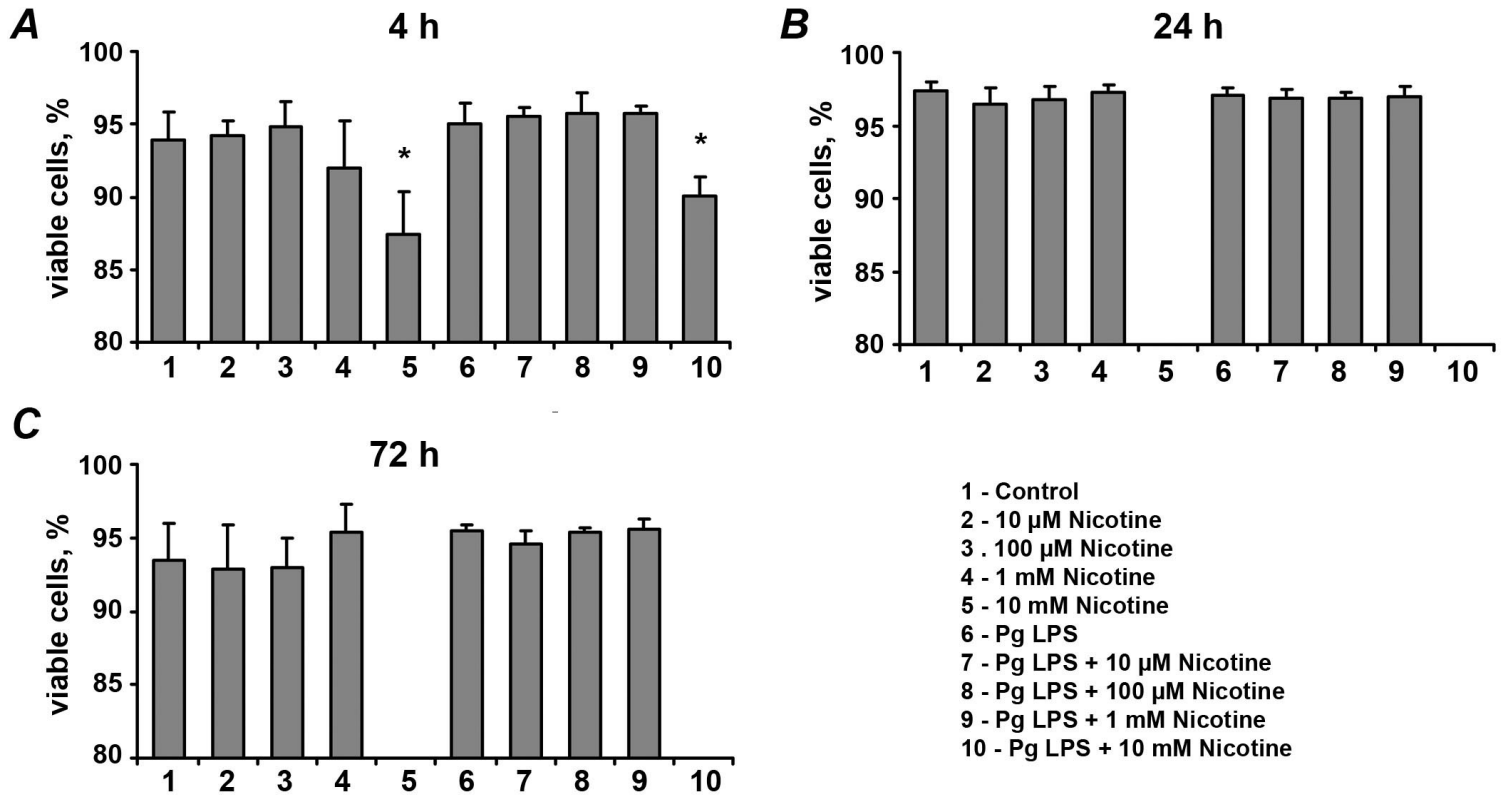
Effect of nicotine and *P. gingivalis* LPS on the proportion of viable HUVECs. HUVECs were stimulated with nicotine (10 µM-10 mM) and/or *P. gingivalis* LPS, and the proportion of viable cells was measured using a flow cytometry apoptosis assay after 4 (A), 24 (B), and 72 (C) h. Viable cells were those negative for annexin V and propidium iodide. Data are presented as mean ±SD of three independent experiments. * – significantly lower compared to control group, p<0.05.

Figure 2 shows the proportion of viable cells after stimulation with different nicotine concentrations and/or *P. gingivalis* LPS for 4, 24, and 72 h. Viable cells were determined as those which were negative for annexin V and propidium iodide. The proportion of viable cells was not affected by nicotine at concentrations of 10 µM-1 mM at any of the stimulation time points. This was true both in the presence and in the absence of *P. gingivalis* LPS. After 4 h of stimulation with 10-mM nicotine, the proportion of viable cells was significantly lower compared to that of the control group (p<0.05).

### Effect of nicotine on the expression of pro-inflammatory mediators in HUVECs

**Figure 3:**
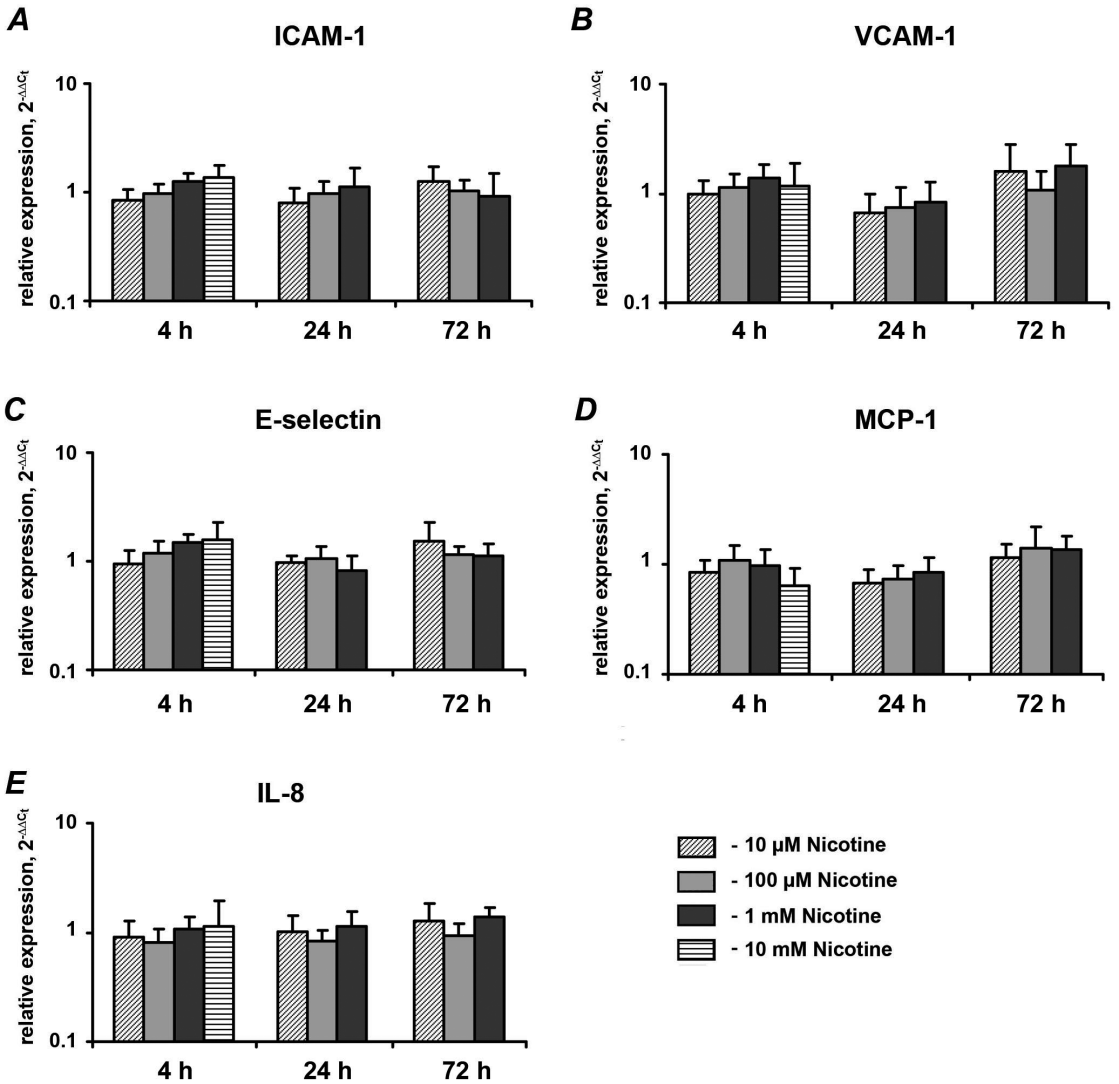
Effect of nicotine on the expression of pro-inflammatory mediators in HUVECs. HUVECs were stimulated with nicotine (10 µM-10 mM), and the expression levels of ICAM-1 (A), VCAM-1 (B), E-selectin (C), MCP-1 (D), and IL-8 (E) were measured by qPCR. GAPDH was used as endogenous control gene. Each value represents mean ±SEM of three independent assays. Non-stimulated HUVECs were used as a control ( = 1). The expression levels of pro-inflammatory mediators were not analyzed after stimulation with 10-mM nicotine for 24 and 72 h because the cells were not viable.

Figure 3 shows the effect of nicotine on the mRNA expression levels of different pro-inflammatory mediators after stimulation for 4, 24, and 72 h. Nicotine at concentration of 10 µM-1 mM had no significant effect on the gene expression levels of ICAM-1 (Fig. 3A), VCAM-1 (Fig. 3B), E-selectin (Fig. 3C), MCP-1 (Fig. 3D), or IL-8 (Fig. 3E) at any of the stimulation time points. Similarly, no significant change in the expression of any of the pro-inflammatory mediators in HUVECs were observed after stimulation with 10-mM nicotine for 4 h.

**Figure 4:**
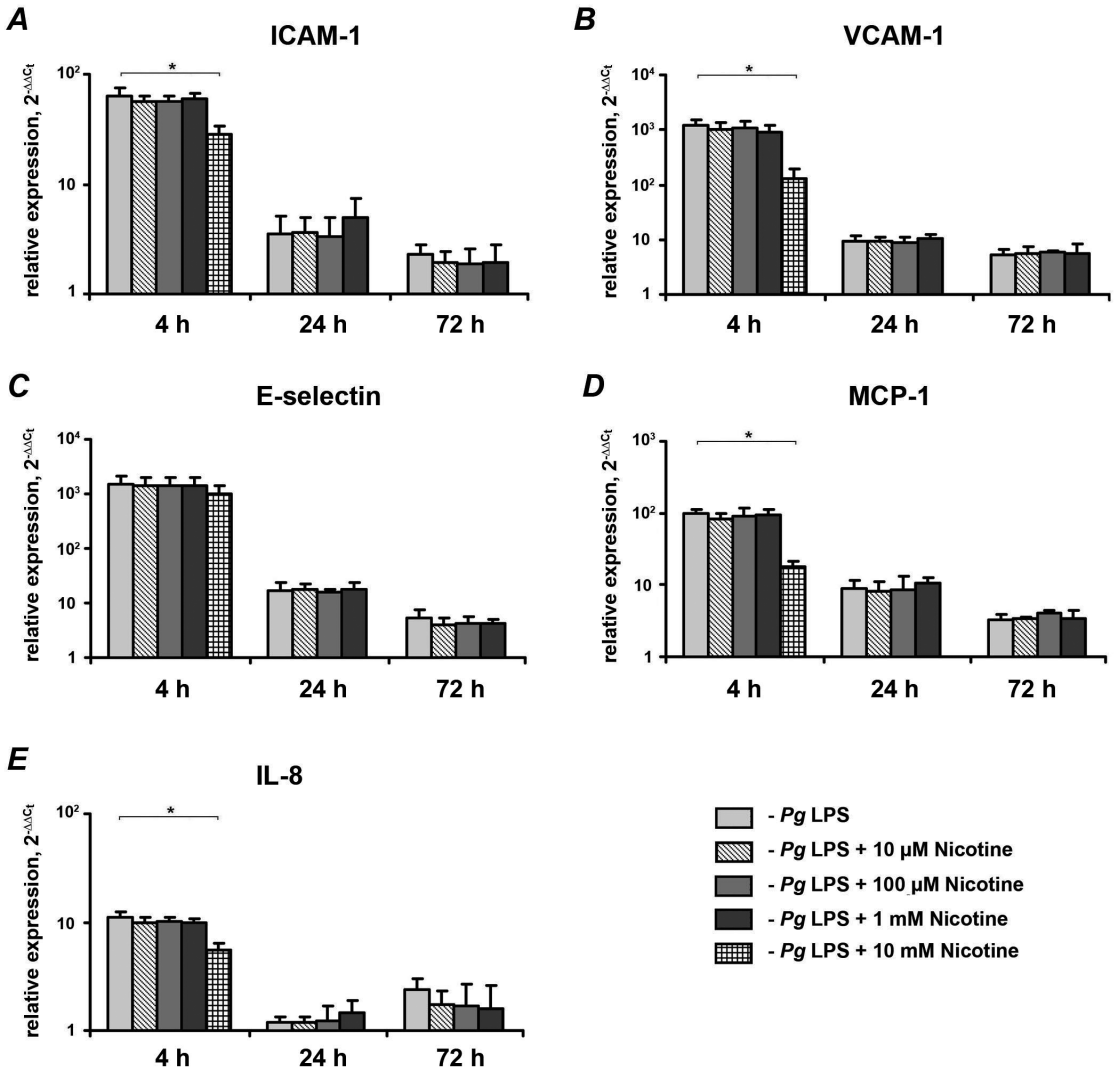
Effect of nicotine on the *P. gingivalis* LPS-induced mRNA expression of pro-inflammatory mediators in HUVECs. HUVECs were stimulated by *P. gingivalis* LPS in the presence or absence of nicotine (10 µM–10 mM), and the expression levels of ICAM-1 (A), VCAM-1 (B), E-selectin (C), MCP-1 (D), and IL-8 (E) were measured by qPCR after 4, 24, and 72 h. GAPDH was used as endogenous control gene. Each value represents mean ±SEM of three independent assays. Non-stimulated HUVECs were used as a control ( = 1). All proteins exhibited significantly higher mRNA expression levels after stimulation with *P. gingivalis* LPS compared to the control group (p<0.05). The expression levels of pro-inflammatory mediators were not analyzed after stimulation with 10-mM nicotine for 24 and 72 h because the cells were not viable. * – significantly different between groups, p<0.05.

**Figure 5:**
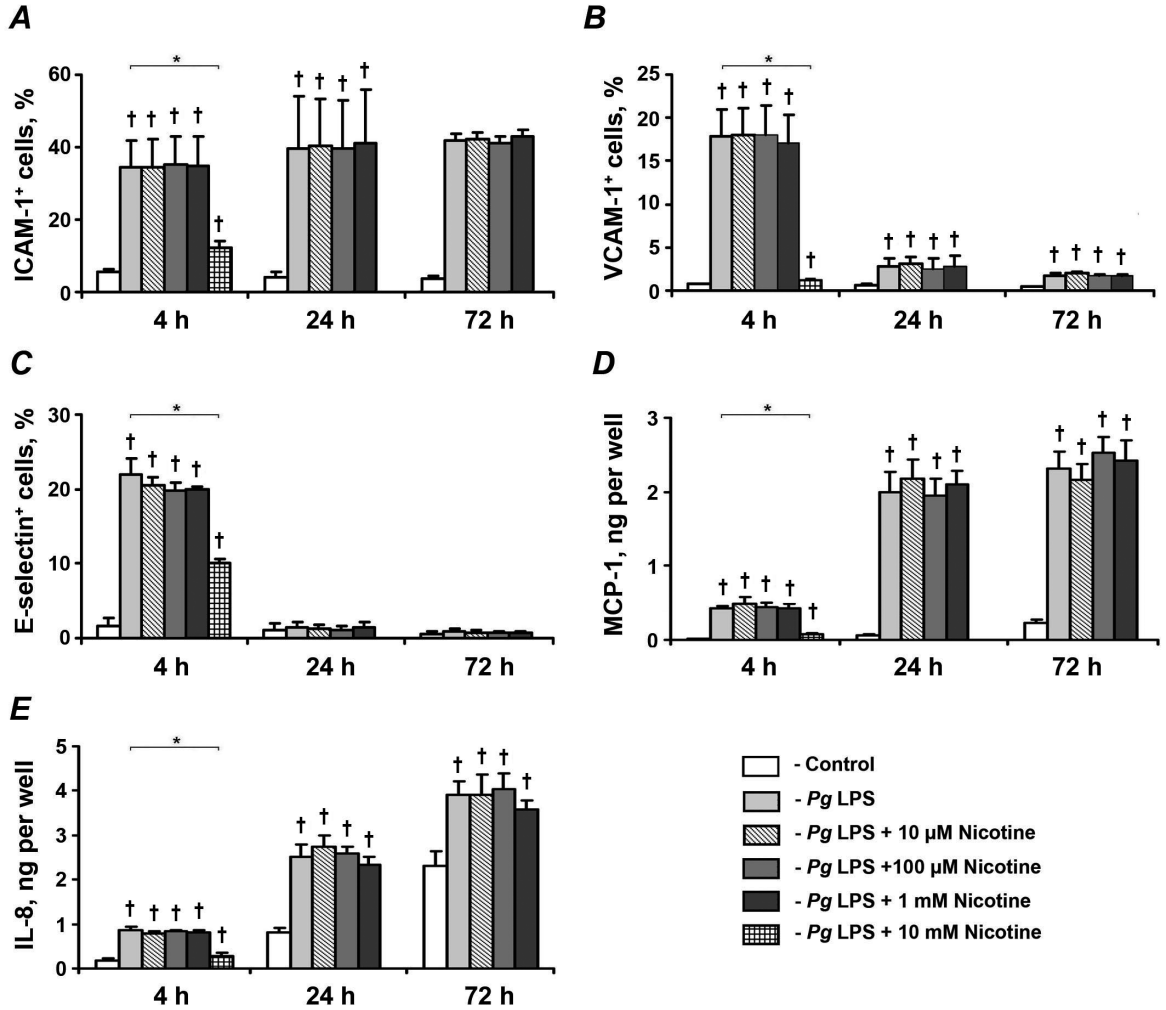
Effect of nicotine on the *P. gingivalis* LPS-induced protein expression of pro-inflammatory mediators in HUVECs. HUVECs were stimulated by *P. gingivalis* LPS in the presence or absence of nicotine (10 µM–10 mM) for 4, 24, and 72 h. After stimulation, the surface expression levels of ICAM-1 (A), VCAM-1 (B), and E-selectin (C) were measured by flow cytometry, and the quantity of MCP-1 (D) and IL-8 (E) in conditioned media was measured by ELISA. Each value represents mean ±SD of three independent assays. Non-stimulated HUVECs were used as a control. The protein expression levels of pro-inflammatory mediators were not analyzed after stimulation with 10-mM nicotine for 24 and 72 h because the cells were not viable. * – significantly different between groups, p<0.05. † – significantly higher compared to controls, p<0.05.

The effect of nicotine on the *P. gingivalis* LPS-induced mRNA and protein expression of pro-inflammatory mediators in HUVECs is shown in Figures 4 and 5, respectively. The largest increase in the mRNA expression levels of ICAM-1 (Fig. 4A), VCAM-1 (Fig. 4B), E-selectin (Fig. 4C), MCP-1 (Fig. 4D), and IL-8 (Fig.4E) was observed after 4 h of stimulation with 1 µg/ml of *P. gingivalis*, whereas the response was substantially diminished after 24 and 72 h of stimulation. Similarly, the largest surface expression levels of ICAM-1 (Fig. 5A), VCAM-1 (Fig. 5B), and E-selectin (Fig. 5C) were observed after 4 h of stimulation. The quantity of MCP-1 and IL-8 in conditioned media (Figs 5D and 5E, respectively) was continuously increased with stimulation time. Nicotine at a concentrations of 10 mM induced a significant decrease of *P. gingivalis* LPS-induced mRNA and protein expression levels of ICAM-1, VCAM-1, MCP-1, and IL-8 after 4 h of stimulation. *P. gingivalis* LPS-induced surface expression of E-selectin was significantly decreased by 10 mM after 4 h of stimulation, whereas the mRNA expression level of E-selectin was not affected. Nicotine at concentrations of 10 µM-1 mM had no significant effect on the *P. gingivalis* LPS-induced expression of pro-inflammatory mediators at any of the stimulation time points.

### Effect of nicotine on HUVEC migration

**Figure 6:**
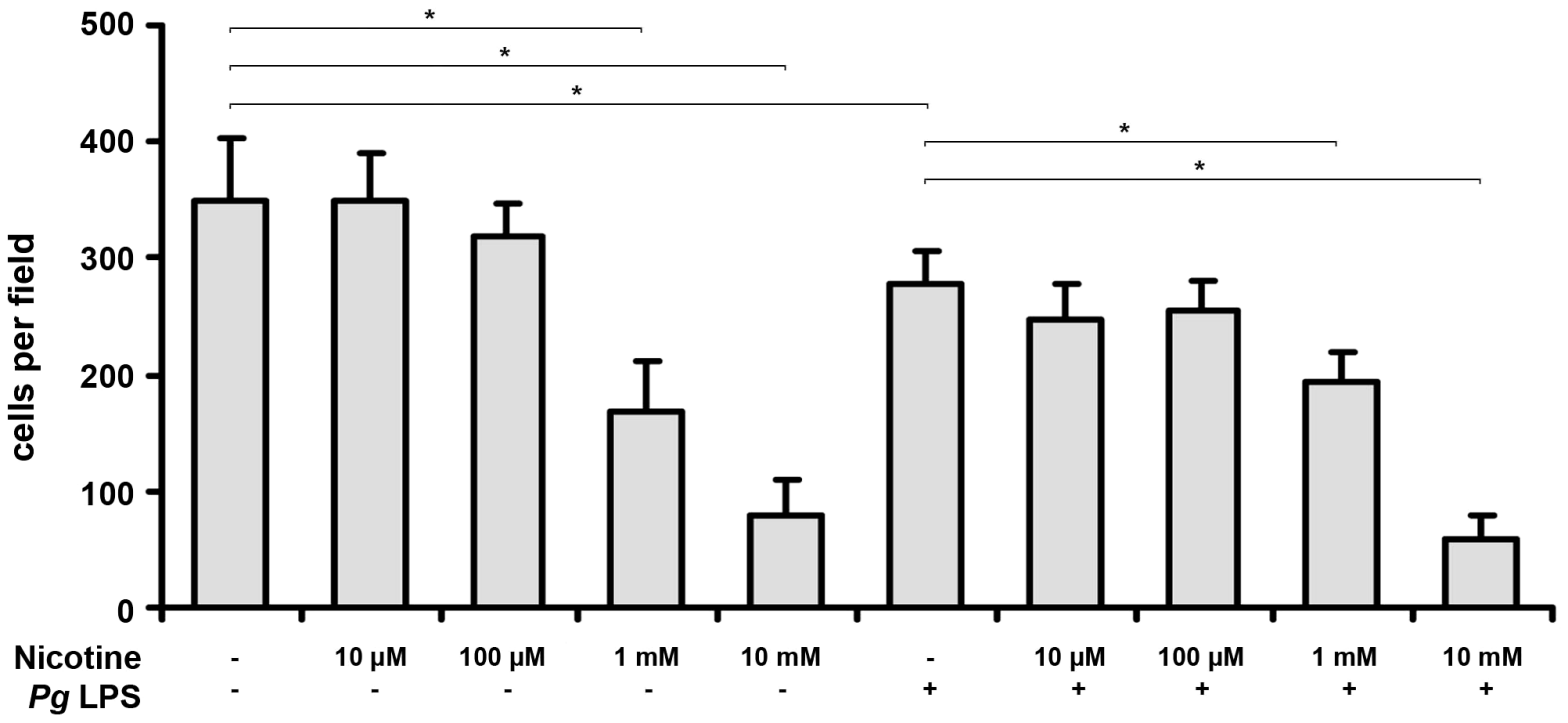
Effect of nicotine and *P. gingivalis* LPS on HUVEC migration measured in the microchemotaxis chamber. HUVEC migration through a polycarbonate membrane with pore size of 8 µm over 8 h was assessed in a 48-well microchemotaxis chamber. The y-axis represents mean ±SD of cells per microscope field of four different wells of one representative experiment. * – significantly different between groups, p<0.05.

Figure 6 shows the number of HUVECs that migrated through the 8-µm polycarbonate membrane in the presence or absence of nicotine at concentrations of 10 µM-10 mM and/or *P. gingivalis* LPS. The number of migrated HUVECs was significantly decreased by nicotine at concentrations of 1–10 mM in a dose dependent manner (p<0.05). This was observed both in the presence and the absence of *P. gingivalis* LPS. *P. gingivalis* LPS induced a significant decrease in the number of migrated cells (p<0.05).

## Discussion

In the present *in vitro* study, we evaluated the effect of nicotine on EC properties under normal and inflammatory conditions. We found that nicotine at concentrations of 10 µM-1 mM had no effect on the proliferation/viability of HUVECs, as measured by MTT assay. At these nicotine concentrations, the proliferation/viability of HUVECs gradually increased with time, suggesting that cells proliferated during the observation period. However, 10-mM nicotine substantially inhibited the proliferation/viability of HUVECs after 4 h of stimulation; after stimulation for longer than 24 h, no viable cells were observed. Thus, it seems that 10-mM nicotine has a cytotoxic effect on HUVECs. Many previous studies also reported that nicotine at high concentrations inhibits EC proliferation and is cytotoxic. However, quantitative differences in estimating the toxicity of nicotine concentrations exist between studies. In particular, a recent study showed that the proliferation/viability of HUVECs is inhibited by nicotine at concentrations higher than 400 µg/ml (∼2.5 mM) [24], which is generally in agreement with our data. In contrast, another study showed that HUVEC proliferation measured by cell count is inhibited by as little as 0.1-µM nicotine [13]. A study on calf ECs showed that cell proliferation measured by DNA synthesis is inhibited by nicotine at concentrations higher than 1 µM [15]. Interestingly, lower nicotine concentrations (<1 µM) might also stimulate proliferation of different ECs [13], [15], [25]. The difference in the experimental conditions, particularly initial cell seeding densities, could help explain the variable results of different studies. In particular, a recent study showed that HUVEC proliferation depends highly on the cell seeding density, and even a small increase of cell density might inhibit proliferation [26]. The diversity of nicotine effects might also be explained by the fact that ECs express a variety of nicotinic acetylcholine receptors which might differently regulate various cellular functions [27].

We further investigated the effect of nicotine on the expression of several factors associated with inflammation, namely ICAM-1, VCAM-1, E-selectin, MCP-1, and IL-8, in the presence and absence of *P. gingivalis* LPS, which was used to model the conditions of periodontal inflammation. An increased expression of adhesion molecules in ECs is tightly associated with the progression of atherosclerosis [28]. In particular, E-selectin mediates the “rolling” of leukocytes along the endothelium, which is the reversible first interaction between endothelial and immune cells, whereas ICAM-1 and VCAM-1 are involved in the firm attachment of leukocytes to the endothelium and trans-endothelial migration [29]. MCP-1 and IL-8 are chemoattractants that induce the migration of neutrophils and monocytes, respectively, to the site of inflammation and promote the development of acute inflammation [30], [31]. In this study, nicotine had no effect on the expression of any pro-inflammatory mediators in the absence of *P. gingivalis* LPS. A recent study showed that nicotine at concentrations of 100 µg/ml (∼0.62 mM) had no effect on the on the production of IL-8 by HUVECs and slightly inhibited MCP-1 production [24]. Another study found that mRNA expression levels of VCAM-1 in human coronary artery ECs were slightly increased by a factor of about 1.6 in response to stimulation with 10-µM nicotine [32]. Finally, the surface expression of ICAM-1 and VCAM-1 in HUVECs was slightly increased by stimulation with 10-µM nicotine [33]. Thus, it might be concluded that the expression of pro-inflammatory mediators in HUVECs is only slightly—if at all—influenced by nicotine.

Stimulation of HUVECs with *P. gingivalis* LPS resulted in a significant increase in the expression of all pro-inflammatory mediators. This was observed at both mRNA and protein levels and is in agreement with previous reports [16]–[19], [22], [34]. The maximal response to *P. gingivalis* LPS was observed 4 h after stimulation, which is also in agreement with previous studies showing that maximal response of ECs to LPS is usually within several hours after stimulation [16], [35]–[37]. It should be also considered that, at the late stages, the autocrine response of HUVECs to produce cytokines might play an important role and interfere with the reaction to LPS. We found that nicotine in concentrations of 10 µM-1 mM did not influence the response of HUVECs to *P. gingivalis* LPS. However, nicotine at a concentration of 10 mM significantly decreased the expression of pro-inflammatory mediators induced by *P. gingivalis* LPS stimulation, which could be related to nicotine cytotoxicity. Interestingly, no significant effect of 10-mM nicotine on the expression of adhesion molecules was found in the absence of *P. gingivalis* LPS stimulation (Fig. 3). Therefore, one can assume that nicotine could influence pro-inflammatory pathways in HUVECs, which are activated upon *P. gingivalis* LPS stimulation. The effect of nicotine on the inflammatory response in HUVECs was also previously investigated. Patton et al showed that pre-incubation of human coronary artery ECs with 1-µM nicotine reduced the expression of IL-8 and MCP-1 in response to LPS [38]. Similarly, Saeed et al showed that nicotine in micromolar concentrations inhibited the expression of ICAM-1 in human microvascular ECs in response to stimulation with tumor necrosis factor α [39]. In contrast to these studies, we observed that only millimolar concentrations of nicotine were able to diminish inflammatory response of HUVECs to LPS. The reason for this discrepancy is not clear. Theoretically, it could be due to heterogeneity in the expression of nicotine receptors in different ECs [27], but this question needs to be investigated further.

Migration of ECs is an important process involved in angiogenesis and wound healing. We found that nicotine at concentrations of 1–10 mM inhibited EC migration in the presence and absence of *P. gingivalis*. The inhibitory effect of 10-mM nicotine on HUVEC migration could be at least partially explained by nicotine cytotoxicity. However, 1-mM nicotine was not cytotoxic as it did not affect HUVEC viability and apoptosis. Previous studies reported that nicotine at a concentration of about 1 µM stimulated EC migration [13], [40], [41]. Another study showed that nicotine at a concentration of 20 µg/ml (∼123.2 µM) had no effect on EC migration [42], which is in agreement with our observations. We can thereby conclude that the effect of nicotine on EC migration depends on its concentration.

Nicotine is the major component of tobacco smoke, and its presence in the body fluids of smokers is significantly increased compared to non-smokers. Previous studies showed that the serum levels of nicotine in smokers achieve near a 1-µM concentration [43]. In contrast, nicotine levels in the saliva of smokers may reach millimolar concentrations [44]. Similar nicotine levels were also assumed to be in respiratory system tissues of smokers [45]. Our study showed that inhibition of HUVEC proliferation, migration, and inflammatory response is induced by millimolar concentrations of nicotine. Therefore, it seems that the effects described in the present study might be physiologically important for the oral cavity but not as much on the systemic level.

A decrease of EC proliferation and migration *in vitro* might suggest less vascularization and impaired wound healing *in vivo*, which is supported by numerous clinical studies (for review, see [46]). For mimicking inflammation *in vitro*, we stimulated HUVECs with *P. gingivalis* LPS. Under these conditions, nicotine in millimolar concentrations attenuated the inflammatory response of HUVECs. A decreased release of chemoattractants MCP-1 and IL-8 following nicotine stimulation might be associated with a decreased requirement to attract leukocytes to sites of inflammation. Similarly, a decreased expression of adhesion molecules ICAM-1, VCAM-1, and E-selectin might be associated with decreased infiltration into the periodontal tissue. Thus, our data suggest that nicotine might decrease periodontal inflammation *in vivo*. This is supported by clinical studies showing that smoking periodontal patients have a decreased inflammatory response and gingival bleeding compared to non-smoking patients (for review, see [47], [48]).

In conclusion, our data show that the functional properties of ECs are affected by millimolar concentrations of nicotine, which are similar to that observed in the oral cavity of smokers. In contrast, micromolar concentrations of nicotine, which are usually present in the serum of smokers, had no influence on the functional properties of ECs. Nicotine at millimolar concentrations inhibited proliferation and migration of ECs as well as their response to periodontal pathogens. These effects of nicotine might be associated with decreased bleeding indices observed in smoking periodontitis patient.
